# The Effects of Daily-Living Risks on Breast Cancer-Related Lymphedema

**DOI:** 10.1245/s10434-024-15946-x

**Published:** 2024-08-01

**Authors:** Mei Rosemary Fu, Bowen Liu, Jeanna Mary Qiu, Yuanlu Sun, Deborah Axelrod, Amber Guth, Stephanie Korth, Howard L. Kremer, Yao Wang

**Affiliations:** 1 School of Nursing and Health Studies, University of Missouri –Kansas City, Kansas City, MO USA; 2 Division of Computing, Analytics, and Mathematics, School of Science and Engineering, University of Missouri –Kansas City, Kansas City, MO USA; 3 Harvard Medical School, Boston, MA USA; 4 College of Nursing/438 CNB, University of Iowa, Iowa City, IA USA; 5 Department of Surgery, New York University School of Medicine, NYU Perlmutter Cancer Center, New York, NY USA; 6 Advanced Practiced Registered Nurse for the Breast Center at University Health Kansas City, University Health – UMKC Health Sciences District, Kansas City, MO USA; 7 University Health – UMKC Health Sciences District, Kansas City, MO USA; 8 Electrical and Computer Engineering and Biomedical Engineering, New York University Tandon School of Engineering, Brooklyn, NY USA

**Keywords:** Breast cancer, Lymphedema, Risks, Daily-living, Skin trauma, Infection, Skin care

## Abstract

**Background:**

Conventional advice to reduce the risk of breast cancer-related lymphedema (BCLE) suggests avoidance of daily-living risks, and limited research has investigated these risks.

**Objective:**

This study aimed to examine the occurrence, patterns, and effects of daily-living risks on BCLE.

**Methods:**

A cross-sectional design was used to collect data from 567 patients at a metropolitan cancer center in the United States. The *Lymphedema Risk-Reduction Behavior Checklist* was used to assess the occurrence of 11 daily-living risks. Descriptive, regression, and factor analyses were performed.

**Results:**

Significant odds of BCLE were associated with infection (odds ratio [OR] 2.58, 95% confidence interval [CI] 1.95–3.42), cuts/scratches (OR 2.65, 95% CI 1.97–3.56), sunburn (OR 1.89, 95% CI 1.39–3.56), oil splash or steam burns (OR 2.08, 95% CI 1.53–3.83), and insect bites (OR 1.59, 95% CI 1.18–2.13). The daily-living risks were clustered into factors related to skin trauma and carrying objects. Skin trauma risk was significantly associated with BCLE (*B* = 0.539, *z* = 3.926, OR 1.714, 95% CI 1.312–2.250; *p* < 0.001). Having three, four, or five skin trauma risks significantly increased the odds of BCLE to 4.31, 5.14, and 6.94 times, respectively. The risk of carrying objects had no significant or incremental effects on BCLE.

**Conclusion:**

Complete avoidance of daily-living risks is challenging given 52.73% of patients incurred more than five daily-living risks. Our study findings underscore the importance of ‘what to do’ strategies to minimize infection and skin trauma.

Breast cancer-related lymphedema (BCLE) is a syndrome of swelling, pain, and other symptoms related to lymph fluid accumulation following breast cancer treatment.^1–4^ BCLE continues to be one of the most common and debilitating long-term adverse effects of breast cancer treatment that negatively impacts patients’ quality of life and daily living functions.^1–7^ Each year, about 300,000 women are diagnosed with new cases of invasive breast cancer in the United States (US).^8^ Advances in breast cancer diagnosis and treatment have helped over 90% of women treated for breast cancer to achieve 5-year survival.^8^ The increased rate and length of breast cancer survival has made it imperative for clinical practice to focus on mitigating the long-term and debilitating adverse effects of cancer treatment, including BCLE. Among more than 4 million women treated for breast cancer in the US, at least 5.6% of those who have undergone sentinel lymph node biopsy and about 20% of those who have undergone axillary lymph node dissection are affected by BCLE.^1–3,5^ Reducing the risk of BCLE is essential as patients are at a lifetime risk of BCLE.

Research evidence regarding risk factors of BCLE related to cancer and its treatment has been supported by extensive studies with high consistency, including late cancer stage, axillary lymph node dissection, number of lymph nodes removed, mastectomy, radiotherapy, and chemotherapy.^9^ Studies also identified modifiable lifestyle risk factors of BCLE, such as high body mass index (BMI) and cardiovascular conditions.^9–12^ While more research is needed, emerging research demonstrates that risks related to medical procedures, including skin punctures, injections, and blood pressure measurements, on the ipsilateral limb may not increase the risk of BCLE.^13–15^

The conventional approach to reducing the risk and preventing exacerbation of BCLE often involves recommendations for patients to avoid certain daily-living risks, including using the affected arm and hand to lift objects, carry a shoulder bag, avoiding infection, sunburn, cutting cuticles, oil splash, and steam burn.^13–15^ Patients with BCLE often express emotional distress related to the avoidance of daily living activities that may increase the risk and exacerbation of BCLE.^16^ Many clinicians recognize the challenges faced by patients to avoid the aforementioned daily-living risks and are often reluctant to impose this significant burden on patients.^14^ Despite the recommendations to avoid daily-living risks of BCLE, to date there is a paucity of data to validate the effects of the daily-living risks on BCLE. The overall goal of our study was to examine the effects of daily-living risks on BCLE. Specifically, we aimed to (1) examine the occurrence and patterns of daily-living risks (e.g., having infection, cuts or scratches, sunburn, oil splash or steam burns, insect bites, pet scratches in the affected arm or hand, or cutting cuticles of the affected hand; using the affected limb to carry or lift heavy objects, carry a shoulder bag, carry groceries, or lift weights); and (2) to explore the effects of daily-living risks on BCLE. We followed the Strengthening the Reporting of Observational Studies in Epidemiology (STROBE) guidelines to report the results of this study.

## Materials and Methods

### Study Design

This study was conducted as part of a larger cross-sectional and observational study that primarily focused on using machine learning methods to detect and diagnose BCLE (1R01CA214085-01).

### Ethical Considerations

This study (IRB #16-01665) was approved by the Institutional Review Board of a metropolitan cancer center in New York City, NY, USA. Written informed consents were obtained from all participants.

### Setting

This study was conducted in a large metropolitan and National Cancer Institute-designated comprehensive cancer center in New York City, NY, USA.

### Sample Size and Participants

The eligible participants were women older than 21 years of age who (1) had completed acute treatment (i.e., surgery, radiation, and chemotherapy) more than 3 months before enrollment; and (2) had no sign of metastatic disease, recurrence, or any prior lymphatic disease. Between December 2016 and March 2020, 570 breast cancer patients were recruited. This sample size was originally calculated for machine learning methods to detect and diagnose BCLE. Participants were excluded from this study if the data regarding daily-living risks were not complete. Ultimately, 567 participants were included in this study.

### Measures and Variables

#### Breast Cancer-Related Lymphedema (BCLE) Risks

The Lymphedema Risk-Reduction Behavior (LRRB) checklist was used to assess the occurrence of 11 daily-living risks: having infection, cuts or scratches, sunburn, oil splash or steam burns, insect bites, pet scratches in the affected arm or hand, or cutting cuticles of the affected hand, and using the affected limb to carry or lift heavy objects, carry a shoulder bag, carry groceries, or lift weights.^17,18^ For each risk, participants were asked to answer whether the risk had occurred or not and, if so, how many times during the last 3 months.

#### BCLE

The presence of BCLE was confirmed for participants if the following criteria were met: (1) patients self-report of being diagnosed with and treated for BCLE; and (2) medical record review to validate the patients’ BCLE status.

### Clinical and Demographic Variables

Clinical information was retrieved from medical records and included types of breast cancer surgery, types of adjuvant therapy (e.g., chemotherapy, hormonal therapy, radiotherapy), lymph node procedures, and years elapsed since breast cancer treatment. Demographic information included age, education, marital status, employment status, ethnicity, and financial status.

#### Data Analysis

Data analyses were performed using R software version 4.3.1 (The R Foundation for Statistical Computing, Vienna, Austria). Means, standard deviations (SD), and ranges were used to summarize continuous variables, while frequencies and proportions were used to summarize categorical variables. To measure the precision of estimates, confidence intervals (CI) were estimated. The Chi-square test for independence was used to assess the association between each daily-living risk and BCLE, and the odds ratio (OR) was used to quantify the effect of each daily-living risk on BCLE.

A factor analysis using varimax rotation methods was performed to further examine the structural patterns of daily-living risks. A significant Bartlett’s test of sphericity (Chi-square [55] = 848.103; *p* < 0.001) and Kaiser–Meyer–Olkin (KMO) value of 0.72 suggested that the data were appropriate for factor analysis. The initial factor analysis was performed on all 11 daily-living risks, resulting in two factors. The daily-living risks of infection and cutting cuticles did not load on either of the two factors and were excluded from the final factor analysis.

A multivariate logistic regression model was used to assess the effects of daily living risks. The estimated OR with corresponding 95% CI was obtained to provide quantitative summaries of the effects while controlling for the demographic and clinical variables. The t-test was used to assess the significance of the model parameters; McFadden’s *R*^2^ and likelihood ratio tests were used to check the goodness-of-fit of the logistic regression model; and Fisher’s exact test was used to assess the associations between BCLE and the number of daily-living risks. Logistic regression models were computed to evaluate the incremental effects of number of daily-living risks on BCLE. All statistical tests were conducted with a significance level of *α* = 0.05.

## Results

### Participant Characteristics

Table 1 presents the demographic and clinical characteristics of the 567 participants. The mean age of all participants was 58.19 years (SD 11.32, range 26–85). Among all participants, 58.55% were married or partnered, 73.19% had a bachelor’s degree or graduate degree, and 64.02% were employed. 75.59% of participants were White and 24.51% were non-White. Regarding financial status, only 7.58% reported financial difficulties of not having enough to make ends meet.

**Table 1:** Demographic and clinical characteristics

Categorical variables [n (%)]	All samples [*N* = 567]
*Financial status*	
Financial difficulties	43 (7.58)
No financial difficulties	524 (92.42)
*Education*	
Associate degree or less	152 (26.81)
Bachelor's degree	215 (37.92)
Graduate degree	200 (35.27)
*Marital status*	
Married/partnered	332 (58.55)
Single/widowed	235 (41.45)
*Ethnicity*	
White	415 (73.19)
Non-White	152 (26.81)
*Employment status*	
Currently employed	363 (64.02)
Not employed	204 (35.98)
*Diagnosis of breast cancer-related Lymphedema (BCLE)*	
Yes	133 (23.46)
No	434 (76.54)
*Chemotherapy*	
Yes	363 (64.02)
No	204 (35.98)
*Radiotherapy*	
Yes	408 (71.96)
No	159 (28.04)
*Hormonal therapy*	
Yes	474 (83.60)
No	93 (16.40)
*Sentinel lymph node biopsy*	
Yes	497 (87.65)
No	70 (12.35)
*Axillary lymph node dissection*	
Yes	335 (59.08)
No	232 (40.92)
*Lumpectomy*	
Yes	268 (47.27)
No	299 (52.73)
*Mastectomy*	
Yes	299 (52.73)
No	268 (47.27)
**Numerical variables [mean ± SD (range)]**	
Age, years	58.19 ± 11.32 (26–85)
Years elapsed since last breast cancer treatment	5.11 ± 5.44 (0–43)
Number of lymph nodes removed	8.63 ± 8.01 (1–40)

Data are expressed as *n* (%) unless otherwise specified

*SD* standard deviation

Regarding clinical characteristics, 64.02% underwent chemotherapy, 71.96% underwent radiotherapy, 83.60% underwent hormonal therapy, 47.27% underwent lumpectomy, and 52.73% underwent mastectomy. In terms of lymph node procedures, 59.08% underwent axillary lymph node dissection and 87.65% underwent sentinel lymph node biopsy. The mean number of years elapsed since the last cancer treatment was 5.11 (SD 5.44, range 0–43), and the mean number of lymph nodes removed was 8.63 (SD 8.63, range 1–40). Of all the participants, 23.46% were diagnosed with BCLE.

### Occurrence of Daily-Living Risks

Regarding the occurrence of daily-living risks, the mean number of risks was 4.71, ranging from 0 to 11, and the median number of risks was 5 of the 11 daily-living risks. Of the 567 patients, 52.73% incurred more than five daily-living risks.

### Effects of Daily-Living Risks on BCLE

Table 2 presents the association between each daily-living risk and BCLE. A significant association existed between BCLE and infection (Chi-square = 38.45, degrees of freedom [*df*] = 1; *p* < 0.001), cuts or scratches (Chi-square = 42.18, *df* = 1; *p* < 0.001), sunburn (Chi-square = 14.65, *df* = 1; *p* < 0.001), oil splash or steam burns (Chi-square = 17.76, *df* = 1; *p* < 0.001), insect bites (Chi-square = 8.84, *df* = 1; *p* = 0.003), and carrying a shoulder bag (Chi-square = 14.79, *df* = 1; *p* < 0.001).

**Table 2:** Daily-living risks between patients with and without a diagnosis of breast cancer-related lymphedema

	All samples [*N* = 567]	BCLE [*n* = 133]	No BCLE [*n* = 434]	Chi-square	df	*p*-Value
*Skin infection*						
Not occurred	454 (80.07)	81 (60.90)	373 (85.94)	38.45	1	< 0.001
Occurred	113 (19.93)	52 (39.10)	61 (14.06)			
*Cuts or scratches*						
Not occurred	377 (66.49)	57 (42.86)	320 (73.73)	42.18	1	< 0.001
Occurred	190 (33.51)	76 (57.14)	114 (26.27)			
*Sunburn*						
Not occurred	459 (80.95)	92 (69.17)	367 (84.56)	14.65	1	< 0.001
Occurred	108 (19.05)	41 (30.83)	67 (15.44)			
*Oil splash or steam burns*						
Not occurred	484 (85.36)	98 (73.68)	386 (88.94)	17.76	1	< 0.001
Occurred	83 (14.64)	35 (26.32)	48 (11.06)			
*Insect bites*						
Not occurred	358 (63.14)	69 (51.88)	289 (66.59)	8.84	1	0.003
Occurred	209 (36.86)	64 (48.12)	145 (33.41)			
*Pet scratches*						
Not occurred	474 (83.60)	104 (78.20)	370 (85.25)	3.20	1	0.073
Occurred	93 (16.40)	29 (21.80)	64 (14.75)			
*Cut cuticles*						
Not occurred	301 (53.09)	76 (57.14)	225 (51.84)	0.94	1	0.331
Occurred	266 (46.91)	57 (42.86)	209 (48.16)			
*Carry or lift heavy objects*						
Not occurred	107 (18.87)	25 (18.80)	82 (18.89)	<0.001	1	1
Occurred	460 (81.13)	108 (81.20)	352 (81.11)			
*Carry shoulder bag*						
Not occurred	177 (31.22)	60 (45.11)	117 (26.96)	14.79	1	< 0.001
Occurred	390 (68.78)	73 (54.89)	317 (73.04)			
*Carry groceries*						
Not occurred	75 (13.23)	11 (8.27)	64 (14.75)	3.17	1	0.075
Occurred	492 (86.77)	122 (91.73)	370 (85.25)			
*Lift weights*						
Not occurred	300 (52.91)	73 (54.89)	227 (52.30)	0.18	1	0.672
Occurred	267 (47.09)	60 (45.11)	207 (47.70)			

Data are expressed as *n* (%)

*BCLE* breast cancer-related lymphedema, *df* degrees of freedom

**Fig. 1:**
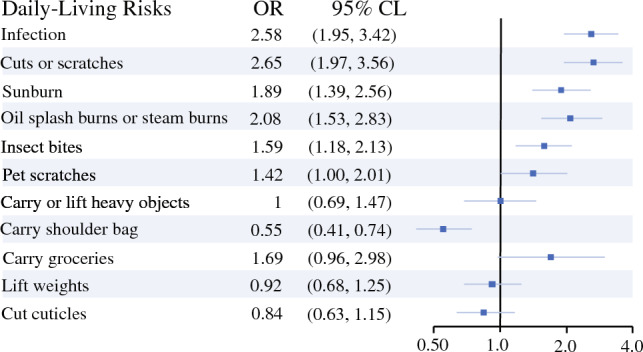
Odds ratio of daily-living risks on the diagnosis of breast cancer-related lymphedema. *OR* odds ratio, *CI* confidence interval

The ORs were computed to quantify the effect of each daily-living risk on BCLE (Fig. 1). Significant odds of BCLE were associated with infection (OR 2.58, 95% CI 1.95–3.42), cuts and scratches (OR 2.65, 95% CI 1.97–3.56), sunburn (OR 1.89, 95% CI 1.39–3.56), oil splash or steam burns (OR 2.08, 95% CI 1.53–3.83), insect bites (OR 1.59, 95% CI 1.18–2.13), and carrying a shoulder bag (OR 0.55, 95% CI 0.41–0.74); however, no significant odds were associated with carrying or lifting heavy objects, carrying groceries, lifting weights, and cutting cuticles.

### Patterns and Effects of Daily-Living Risks on BCLE

**Fig. 2:**
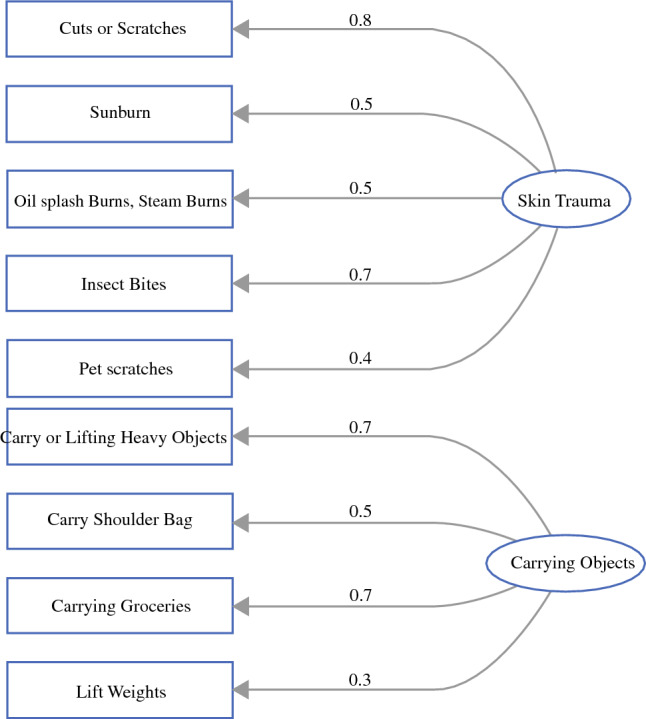
Factor structures for daily-living risks of breast cancer-related lymphedema

Figure 2 presents the path diagram of two factors extracted from the final factor analysis. The first factor was labeled as ‘Skin Trauma’, since this factor comprised the daily-living risks of cuts and scratches, sunburn, oil splashes or steam burns, insect bites, and pet scratches. The second factor was termed as ‘Carrying Objects’, since this factor was involved in daily-living risks of using the affected arm and hand to carry or lift heavy weights, carry a shoulder bag, carry groceries, and lifting weights. The factor of skin trauma explained 54% of the variance, and the factor of carrying objects explained 46% of the variance.

Table 3 presents the multivariate logistic regression models. Daily-living risk of skin trauma was significantly associated with BCLE (*B* = 0.539, *z* = 3.926, OR 1.714, 95% CI 1.312–2.250; *p* < 0.001) along with infection (*B* = 0.582, *z* = 3.851, OR 1.789, 95% CI 1.336–2.420; *p* < 0.001) and radiation therapy (*B* = 0.719, *z* = 2.310, OR 2.052, 95% CI 1.127–3.828; *p* = 0.021). No significant association was found between BCLE and the daily-living risk of carrying objects.

Table 4 presents the association between BCLE and the number of daily-living risks of skin trauma and carrying objects. The number of skin trauma risks was significantly associated with BCLE (*t* = 5.75, mean 1.20 ± SD 1.40, range 0–5, *df* = 197.73; *p* < 0.001), while the number of ‘carrying objects’ risks had no significant association (*t* = 1.27, mean 2.84 ± SD 1.15, range 0–4, *df* = 228.65; *p* = 0.204). Logistic regression models revealed significant and incremental effects of number of skin trauma risks on BCLE. Compared with patients with no skin trauma risks, having three, four, or five skin trauma risks significantly increased the odds of BCLE to 4.31, 5.14, and 6.94 times, respectively (*p* < 0.001). Compared with patients with no risks of carrying objects, there were no significant and incremental odds of BCLE (*p* = 0.060).

**Table 3:** Multivariate logistic regression on diagnosis of breast cancer-related lymphedema [*N* = 567]

Predictors	B	SE	OR	95% CI	*z*-value	*p*-value
*Education*						
Bachelor's degree	0.111	0.296	1.118	(0.627–2.007)	0.376	0.707
Graduate degree	−0.378	0.312	0.686	(0.371–1.264)	−1.210	0.226
Associate degree or less	–	–	–	–	–	–
*Marital status*						
Not married/partnered	0.357	0.241	1.429	(0.892–2.296)	1.484	0.138
Married	–	–	–	–	–	–
*Employment status*						
Yes	−0.222	0.259	0.801	(0.482–1.335)	−0.856	0.392
No	–	–	–	–	–	–
*Ethnicity*						
White	0.024	0.288	1.024	(0.586–1.815)	0.082	0.934
Non-White	–	–	–	–	–	–
*Financial status*						
Financial difficulties	−0.079	0.422	0.924	(0.389–2.054)	−0.187	0.852
No difficulties	–	–	–	–	–	–
*Chemotherapy*						
Yes	0.539	0.293	1.714	(0.970–3.077)	1.836	0.066
No	–	–	–	–	–	–
*Radiation*						
Yes	0.719	0.311	2.052	(1.127–3.828)	2.310	0.021
No	–	–	–	–	–	–
*Mastectomy*						
Yes	0.262	0.537	1.299	(0.429–3.387)	0.488	0.626
No	–	–	–	–	–	–
*Lumpectomy*						
Yes	−0.517	0.518	0.596	(0.203–1.574)	−0.999	0.318
No	–	–	–	–	–	–
*Sentinel lymph node biopsy*						
Yes	−0.178	0.323	0.837	(0.446–1.590)	−0.551	0.582
No	–	–	–	–	–	–
*Axillary lymph node dissection*						
Yes	0.366	0.332	1.442	(0.751–2.777)	1.100	0.271
No	–	–	–	–	–	–
*Hormonal therapy*						
Yes	−0.192	0.315	0.826	(0.450–1.553)	−0.609	0.543
No	–	–	–	–	–	–
*Skin infection*						
Occurred	0.582	0.151	1.789	(1.336–2.420)	3.851	<0.001
Not occurred	–	–	–	–	–	–
(Intercept)	−3.722	1.117	0.024	(0.003–0.213)	−3.332	<0.001
Age	0.019	0.013	1.019	(0.994–1.044)	1.019	0.142
Number of lymph nodes removed	0.026	0.018	1.026	(0.991–1.063)	1.463	0.144
Years since last breast cancer treatments	0.033	0.021	1.033	(0.991–1.078)	1.536	0.125
Factor 1: Skin trauma	0.539	0.137	1.714	(1.312–2.250)	3.926	<0.001
Factor 2: Carrying objects	0.134	0.149	1.143	(0.860–1.547)	0.895	0.371

*SE* standard error, *OR* odds ratio, *CI* confidence interval

## Discussion

Our study found that 52.73% of the 567 participants incurred more than five daily-living risks. It is clear that complete avoidance of daily-living risks is challenging. This finding provides insight into the avoidance approach to BCLE risk reduction education. A paradigm shift in BCLE risk reduction is imperative to empower, rather than inhibit, how patients live their lives by emphasizing ‘what to do’, rather than ‘what to avoid’.

Skin trauma poses a risk of infection.^15^ Our study identified significant associations between BCLE and daily-living risks of infection and skin trauma (i.e., cuts or scratches, sunburn, oil splash or steam burns, and insect bites). Our study also found that infection had 2.58 times odds of BCLE. This finding is supported by previous studies that patients with a history of infections such as wound infection, lymphangitis, and cellulitis had a higher risk of developing and exacerbating lymphedema.^19,20^ Interestingly, sunburn, identified as a risk in our study, has not been explored in prospective research or larger patient samples. Li et al.^21^ reported a case of lymphedema induced by prolonged sun exposure 11 years after breast cancer surgery. The patient, who had undergone a right breast mastectomy, chemotherapy, and radiation, had no signs of lymphedema for 11 years. She experienced swelling, pain, and chest wall infection after direct sun exposure during a 4-day fieldwork project. Our findings corroborate this case study and highlight the importance of preventing sunburn as a BCLE risk reduction strategy. Moreover, the odds of BCLE significantly increased with the number of skin trauma risks: having three, four, or five of the daily-living risks of skin trauma increased the odds of BCLE to 4.31, 5.14, and 6.94 times, respectively. Our study is the first to demonstrate the evidence concerning the incremental effects of skin trauma on BCLE. These findings underscore the importance of implementing strategies to minimize daily-living risks of infection and skin trauma. Skin care strategies that prevent injury are important to prevent infection.^25^ Furthermore, daily skin care to maintain skin hygiene and keep skin moisturized can prevent dry skin that is easy to breakdown and allows bacteria to penetrate the protective skin barrier. Table 5 presents research-based strategies to minimize daily-living risks.^18,22–25^

**Table 4:** Associations between number of daily-living risks and the diagnosis of breast cancer-related lymphedema

Number of daily-living risks related to skin trauma^1^			
Number of risks	BCLE		*p*-Value: <0.001
	No [*n/N* (%)]	Yes [*n/N* (%)]	OR (95% CI)
0	229/434 (52.8)	33/133 (24.8)	1.00
1	75/434 (17.3)	30/133 (22.6)	2.78 (1.58–4,86)
2	62/434 (14.3)	23/133 (17.3)	2.57 (1.40–4.69)
3	37/434 (8.5)	23/133 (17.3)	4.31 (2.28–8.15)
4	27/434 (6.2)	20/133 (15.0)	5.14 (2.58–10.20)
5	4/434 (0.9)	4/133 (3.0)	6.94 (1.57–30.65)

Number of daily-living risks related to carrying objects^2^			
Number of risks	BCLE		*p*-Value: 0.060
	No [*n/N* (%)]	Yes [*n/N* (%)]	OR (95% CI)
0	29/434 (6.7)	6/133 (4.5)	1.00
1	33/434 (7.6)	13/133 (9.8)	1.90 (0.66–6.02)
2	53/434 (12.2)	29/133 (21.8)	2.64 (1.04–7.71)
3	169/434 (38.9)	48/133 (36.1)	1.37 (0.57–3.83)
4	150/434 (34.6)	37/133 (27.8)	1.19 (0.49–3.37)

*BCLE* breast cancer-related lymphedema, *OR* odds ratio, *CI* confidence interval

^1^Five daily-living risks of skin trauma: cuts or scratches, sunburn, oil splash or steam burns, insect bites, and pet scratches

^2^Four daily-living risks of carrying objects: carrying or lifting heavy objects, carrying shoulder bag, carrying groceries, and lifting weights

**Table 5:** Strategies to minimize daily-living risks of breast cancer-related lymphedema^18,22–25^

**What should I do to prevent trauma/injuries in my affected hand and arm?**	
Wear protective gloves while gardening or doing household chores (washing dishes, cleaning, or cooking)	
Wear oven mitts when taking hot pots or casseroles out of the oven	
Apply sunscreen (SPF 30 or more) or wear long-sleeved clothes to prevent sunburn.	
Apply insect repellant or wear an insect-repellant band to prevent insect bites	
Cuticles should be pushed back and kept moist; manicure tools should be sterilized	
**What should I do for daily skin care?**	
Keep skin clean and dry	
Use water-based lotions that help the skin to absorb readily and low-pH lotions that provide an active barrier against infection	
**What should I do when I have cuts or scratches on my affected arm or hand?**	
Wash the area with soap and water	
Apply an antibiotic cream or ointment	
Cover with a clean and dry dressing as needed	
If the area becomes worse, call your doctor	
**What should I do when I have mosquito or insect bites?**	
For itching, apply hydrocortisone ointment	
If the area is red and slightly inflamed, apply an antibiotic ointment	
If the area becomes worse, call your doctor	
**What should I do if I notice any changes in my affected arm or hand?**	
Do not ignore any changes in your affected arm or hand	
If you notice a rash, blisters, redness, increased warmth/heat in the arm, and/or pain, please contact your doctor right away	

*SPF* sun protection factor

Patients are often instructed to avoid or limit strenuous use of the affected arm and hand due to the fear of developing BCLE.^4,14,15^ Our study found no significant associations between daily-living risks of carrying or lifting heavy objects, carrying groceries, and lifting weights. Carrying a shoulder bag on the affected arm was significantly associated with BCLE, with odds of 0.55, indicating that carrying a shoulder bag may have a protective effect on BCLE. It is important to note that our study also found that there was no significant association between the number of ‘carrying objects’ risks and BCLE, as well as incremental effects of the increased number of ‘carrying objects’ risks on BCLE. Over the last decade, studies have justified slow and progressive weightlifting was a safe practice for breast cancer survivors with lymphedema or patients at risk of developing lymphedema.^26,27^ Further research is needed to confirm the association of daily-living risks of carrying objects on BCLE that we observed in this study. Until evidence is further established, patients should be cautioned about daily-living risks of carrying objects that may cause injuries to the affected arm or hand.

### Limitations

The cross-sectional design of this study limits its ability to fully establish a causal relationship between the daily-living risks and BCLE. There may be recall bias in patients’ reports of the occurrence of daily livings risks; for example, patients with BCLE may have been more likely to report skin trauma events. Caution should be exercised regarding the generalizability of the study findings. However, our findings, which were generated from a considerably large sample size and systematic assessment of daily-living risks, are valuable hypotheses to be investigated in future prospective and longitudinal designs to investigate the dynamic relationships between daily-living risks and BCLE.

## Conclusion

Our study provides much-needed evidence regarding the daily-living risks of BCLE that offers insights into targeted strategies to reduce the risk of BCLE. The findings of this study provide evidence that both supports and challenges existing recommendations for daily-living risks of BCLE. Our finding that daily-living risks of infection and skin trauma increase the risk of BCLE supports the fact that patients should be educated on ‘what to do’ strategies to minimize daily-living risks of infection and skin trauma, as shown in Table 5. Until further evidence is established regarding the associations between BCLE and daily-living risks of carrying objects, patients should continue to be cautioned against using their affected arm or hand to carry a shoulder bag or other heavy objects or perform strenuous lifting that may cause trauma or injuries. High-quality, prospective and longitudinal research as well as accurately documented case studies are essentially needed to further validate daily-living risks for BCLE identified in our study.
